# Identification of anoikis-related genes classification patterns and immune infiltration characterization in chronic rhinosinusitis with nasal polyps based on machine learning

**DOI:** 10.3389/fmolb.2025.1624300

**Published:** 2025-08-15

**Authors:** Ziqi Chen, Lingmei Qu, Qing Hao, Shuang Teng, Shuo Liu, Qin Wu, Hongtian Yi, Xianji Shen, Liang Li, Zhaonan Xu, Yanan Sun

**Affiliations:** ^1^ Department of Otolaryngology, Head and Neck Surgery, The Second Affiliated Hospital of Harbin Medical University, Harbin, China; ^2^ Department of Otolaryngology, Head and Neck Surgery, The Fifth Affiliated Hospital of Harbin Medical University, Daqing, China

**Keywords:** chronic rhinosinusitis with nasal polyps, anoikis-related genes, immune infiltration, molecular cluster, machine learning, immunotherapy

## Abstract

**Introduction:**

Chronic rhinosinusitis with nasal polyps (CRSwNP) is characterized by stromal edema, albumin deposition, and pseudocyst formation. Anoikis, a process in which cells detach from the correct extracellular matrix, disrupts integrin junctions, thereby inhibiting improperly proliferating cells from growing or adhering to an inappropriate matrix. Although anoikis is implicated in immune regulation and CRSwNP pathogenesis, its specific mechanistic role remains poorly defined.

**Methods:**

The GSE136825 and GSE179625 datasets were obtained from the GEO database and 338 anoikis-related genes (ARGs) were extracted from the literature and databases. Immune cell infiltration was analysed using the CIBERSORT algorithm. CRSwNP samples were classified via consensus clustering. Key ARGs were identified through machine learning. The diagnostic performance of candidate genes was evaluated using Receiver Operating Characteristic (ROC) analysis. Functional annotation was performed based on Gene Ontology (GO) terms, and pathway enrichment analysis was conducted using the Kyoto Encyclopedia of Genes and Genomes (KEGG) database. Regulatory networks were visualized using NetworkAnalyst and Cytoscape. Experimental validation included quantitative real-time reverse-transcription PCR (qRT-PCR), immunohistochemistry (IHC), and immunofluorescence (IF) in human tissues.

**Results:**

Consensus clustering stratified CRSwNP patients into two distinct anoikis-related clusters. Machine learning identified four key genes: CDH3, PTHLH, PDCD4, and androgen receptor (AR). The nomogram model demonstrated high diagnostic accuracy, with an area under the receiver operating characteristic curve (AUC) >0.90. Immune infiltration analysis revealed differential immune microenvironments between clusters, with AR overexpressed in cluster 1 and PTHLH in cluster 2. Network analysis identified 862 drugs or compounds targeting AR. Experimental validation confirmed consistency between bioinformatics predictions and tissue-level expression patterns.

**Conclusion:**

This study delineates two anoikis-related molecular subtypes of CRSwNP and identifies AR and PTHLH as cluster-specific biomarkers. These findings provide novel insights for personalized therapy, drug screening, and immunomodulatory strategies in CRSwNP.

## 1 Introduction

Chronic rhinosinusitis with nasal polyps (CRSwNP) is a common inflammatory disease with typical symptoms including nasal congestion, olfactory disturbances, facial pressure and rhinorrhoea, which severely affects the health-related quality of life (Stevens et al., 2016; Han et al., 2021). Standard treatment modalities include intranasal corticosteroids, short-course systemic corticosteroids, and nasal surgeries. While short-term systemic corticosteroids reduce polyp size and symptoms, prolonged use carries risks of adverse effects. Furthermore, nasal surgeries are associated with high recurrence rates and potential complications such as scarring or mucosal damage (Xu et al., 2020; Fokkens et al., 2020). However, the precise pathogenesis of CRSwNP remains obscure, with the condition hypothesized to arise from a multifactorial interplay of genetic, topographic, anatomical, molecular, hemodynamic, and immunological factors (Kato et al., 2022). Consequently, there is an urgent need to elucidate its etiological mechanisms and identify novel biomarkers to improve diagnostic accuracy and prognostic evaluation in CRSwNP.

Anoikis, a distinct form of programmed cell death (PCD), is characterized by cell detachment from the extracellular matrix (ECM). This process prevents abnormal proliferation and growth when cells adhere to inappropriate matrices, serving as an apoptosis-linked regulatory mechanism (Taddei et al., 2012). Current studies have explored the intrinsic and extrinsic pathways of anoikis regulation, including integrins, epidermal growth factor receptor (EGFR), TGF-β signaling, NF-κB signaling, hypoxia, reactive oxygen species (ROS), and the Hippo pathway (Adeshakin et al., 2021; Frisch et al., 2013). Research indicates differential expression of TGF-β1 in nasal polyps compared to healthy nasal mucosa, revealing its dual role. While TGF-β1 exerts anti-inflammatory effects by suppressing pro-inflammatory genes and proteins, it also upregulates fibrosis- and angiogenesis-related factors, thereby promoting tissue remodeling and cell proliferation (Balsalobre et al., 2013; Little et al., 2008).

Dysregulation of matrix metalloproteinases (MMPs) and their tissue inhibitors (TIMPs), particularly IL-17A-induced activation of the NF-κB pathway leading to MMP-9 upregulation, is a key driver of pathological tissue remodeling in chronic rhinosinusitis with nasal polyps (CRSwNP) (Watelet et al., 2004; Li et al., 2010; Kostamo et al., 2008; Chen et al., 2018). Additionally, IgE-mediated mast cell activation involves reactive oxygen species (ROS) generation (Wang et al., 2018). Furthermore, excessive upregulation of Hippo pathway components in nasal polyp epithelial cells (NPECs), combined with the effector Yes-associated protein (YAP), promotes epithelial cell proliferation and remodeling in CRSwNP (Deng et al., 2019). Finally, inflammatory factors such as TIMP-1, IL-4, IL-6, and TNF-α may activate caspase cascades in CRSwNP through receptor binding (Wen et al., 2022).

Emerging evidence has validated ARGs as novel biomarkers for various pathologies: in oncology, they facilitate diagnosis of lung adenocarcinoma and hepatocellular carcinoma (Chen et al., 2023; Diao et al., 2023); in cardiovascular medicine, they aid risk stratification for ischemic stroke (Qin et al., 2023). However, the exploration of ARGs’ mechanistic role in CRSwNP pathogenesis remains conspicuously absent, particularly regarding their correlations with cellular dynamics in nasal polyp microenvironments.

**FIGURE 1:**
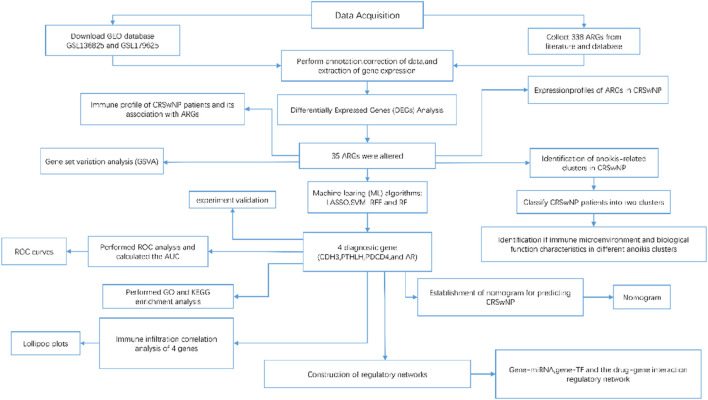
A detailed flow chart about the study of ARGs in CRSwNP.

In this study, we systematically characterized the expression dynamics of ARGs and immune landscape disparities between normal sinonasal mucosa and nasal polyp tissues. By screening anoikis-associated differentially expressed genes (DEGs), we performed molecular subtyping through consensus clustering, which revealed distinct immune infiltration patterns quantified via CIBERSORTx analysis. Functional enrichment analysis demonstrated these DEGs were predominantly involved in epithelial-mesenchymal transition and eosinophil activation pathways. Additionally, we explored the correlation between immune infiltration and regulatory networks involving gene-miRNA, gene-TF, and drug interactions associated with the identified risk genes. Subsequent to this, preliminary experimental verification was conducted within the nasal polyp tissue. The detailed workflow schematic of the current study is illustrated in Figure 1. These findings provide novel insights into anoikis-mediated immunopathological mechanisms in CRSwNP and establish a foundation for precision medicine strategies in clinical management.

## 2 Materials and methods

### 2.1 Data acquisition

We obtained gene expression profiling datasets for two nasal polyp samples associated with chronic rhinosinusitis with nasal polyposis (CRSwNP), specifically GSE136825 (platform GPL20301) and GSE179265 (platform GPL24676), from the NCBI Gene Expression Omnibus (GEO) database. The former dataset was designated as the training set, consisting of 33 control samples and 42 CRSwNP samples, while the latter was used as the validation set, comprising 7 control samples and 27 CRSwNP samples. The raw data from GEO were normalized using the R package “NormalizeBetweenArray.” A total of 338 differentially expressed genes (DEGs) were identified from both published literature and the GeneCards database.

### 2.2 Differentially expressed genes analysis

The R package limma was used to identify differentially expressed genes (DEGs) between normal and CRSwNP samples, with a significance threshold of p < 0.05. DEGs with |log2 fold change| > 1 were selected.

### 2.3 Immune cell infiltration profile

We employed the CIBERSORT algorithm to assess the relative proportions of 22 infiltrating immune cell types based on gene expression, with a significance threshold set at *p* < 0.05. The resulting data was then utilized for further analysis. Group comparisons of immune cell proportions were conducted utilizing the Wilcoxon test. Data visualization, such as histograms, heat maps, and box plots, was created utilizing the “ggplot2” and “vioplot” R packages. Pearson correlation coefficients for each immune cell were calculated using the “corrplot” R package, and the outcomes were illustrated in an accompanying heat map.

### 2.4 Correlation of infiltrating immune cells with ARGs

We computed the correlation coefficient between the expression of ARGs and the percentage of infiltrating immune cells, and visualized the results using the R package “ggplot.” A significance level of *p* < 0.05 was applied to detect any significant correlations.

### 2.5 Construction of unsupervised clusters of anoikis and PCA analysis

We performed unsupervised cluster analysis on ARGs using the “ConsensusClusterPlus” R package to identify distinct anoikis patterns in CRSwNP. The optimal number of subtypes (k) was determined based on the tendency, smoothness of the cumulative distribution function (CDF) curve, consensus score, and consensus matrix. Principal component analysis (PCA) was conducted using the “ggplot2” R package.

### 2.6 Gene set variation analysis

We acquired the “c5.go.symbols” and “c2.cp.kegg.symbols” datasets from the Gene Set Variation Analysis (GSVA) MSigDB database. Subsequently, we employed the R packages “GSVA” and “limma” to assess the modified pathways and biological functions within different clusters linked to ARGs.

### 2.7 Machine learning algorithms

We applied the Least Absolute Shrinkage and Selection Operator (LASSO), Support Vector Machine-Recursive Feature Elimination (SVM-RFE), and Random Forest (RF) algorithms to identify key ARGs among the Differentially Expressed Genes (DEGs) from both normal and CRSwNP samples. These selected genes were then compared using a Venn diagram created with the “VennDiagram” R package. Subsequently, ROC curves were constructed to assess the predictive capacity of these signature genes in the training set, and the Area Under the Curve (AUC) was calculated using the “pROC” and “InpROC” R packages, respectively. The predictive efficacy of these signature genes was further validated in the verification set. Finally, a nomogram based on these signature genes was developed using the “rms” R package.

### 2.8 Gene ontology and Kyoto encyclopedia of genes and genomes analysis

Gene Ontology (GO) and Kyoto Encyclopedia of Genes and Genomes (KEGG) enrichment analyses were conducted on the identified genes using the R package “clusterProfiler” to explore the distinct signaling pathways and potential functions associated with the signature genes. Statistical significance was set at *p* < 0.05.

### 2.9 Correlation of immune-infiltrating cells with signature genes

In the study, we first calculated the correlation coefficients between the expression levels of ARGs and immune infiltrating cells. Subsequently, Spearman’s rank correlation analysis was employed to investigate the association between immune infiltrating cells and the specific genes. Ultimately, Lollipop plots were generated using the R package “ggplot.”

### 2.10 Construction of regulatory networks

Regulatory networks for miRNA diagnostic biomarkers and transcription factor (TF) diagnostic biomarkers were built utilizing characteristic genes with NetworkAnalyst (http://www.networkanalyst.ca). Furthermore, a file containing interactions between drugs and genes was acquired from the drug-gene interaction database (DGIdb) (https://dgidb.genome.wustl.edu/) and imported into Cytoscape software for visualization.

### 2.11 Quantitative real-time reverse transcription PCR

In order to validate the expression of candidate genes in nasal polyps and inferior turbinate tissues (control groups), total RNA was extracted from the nasal polyps and control groups (three samples for each group) using TRIzol Universal reagent (TIANGEN, Beijing, China). The extracted RNA was then reverse transcribed with the PrimeScript RT kit (TaKaRa, Dalian, China). qRT-PCR was performed in triplicate using FastStart Universal SYBR Green Master (ROX) (Roche), with GAPDH serving as an internal control. The relative mRNA levels were calculated using the 2(-Delta Delta CT) method. The Mann-Whitney U test was employed for comparing the two groups of data due to non-normal distribution. The primers utilized for qRT-PCR are listed in Table 1.

**TABLE 1:** Sequences of primers.

Gene	Forward primer 5′-3′	Reverse primer 5′-3′
CDH3	ATCATCGTGACCGACCAGAAT	GACTCCCTCTAAGACACTCCC
PTHLH	ATTTACGGCGACGATTCTTCC	GCTTGGAGTTAGGGGACACC
PDCD4	GCAAAAAGGCGACTAAGGAAAAA	TAAGGGCGTCACTCCCACT
AR	CCAGGGACCATGTTTTGCC	CGAAGACGACAAGATGGACAA

### 2.12 Immunofluorescence

Sections of human sinonasal mucosal tissues, embedded in paraffin at a thickness of 4 μm, were prepared and analyzed to evaluate the expression levels of the target proteins CDH3, PTHLH, PDCD4, and AR.

### 2.13 Immunohistochemistry

For immunohistochemical staining, the paraffin sections were treated with specific primary antibodies. Each slide was randomly examined and photographed in 5 high-power fields of view. Subsequently, the images were exported, and the average optical density value within the field of view was analyzed using Image-ProPlus 6.0 software, with the results being recorded. The characteristic gene expression in nasal polyps was semi-quantitatively assessed using the H-Score formula: $\sum \left(\text{pi}\times i\right)=\left(\text{percentage of weak intensity cells}\times 1\right)+\left(\text{percentage of moderate intensity cells}\times \;2\right)+\left(\text{percentage of strong}\right.$ $\left.\text{intensity cells}\times 3\right)$ . In this formula, “i” indicates the classification of positive cells, where negative cells without coloring are scored as 0 points, weakly positive cells as 1 point (light yellow), moderately positive cells as 2 points (brown-yellow), and strongly positive cells as 3 points (brown-brown). “pi” represents the percentage of positive cells for each grade.

### 2.14 Statistical analysis

Statistical analyses were conducted using R version 4.1.1 and GraphPad Prism 8. Univariate and multivariate logistic regression analyses were employed to evaluate the diagnostic efficacy of the predictive model. All statistical tests were two-tailed, with statistical significance set at *P* < 0.05.

## 3 Results

### 3.1 Expression profiles of ARGs in CRSwNP patients

**FIGURE 2:**
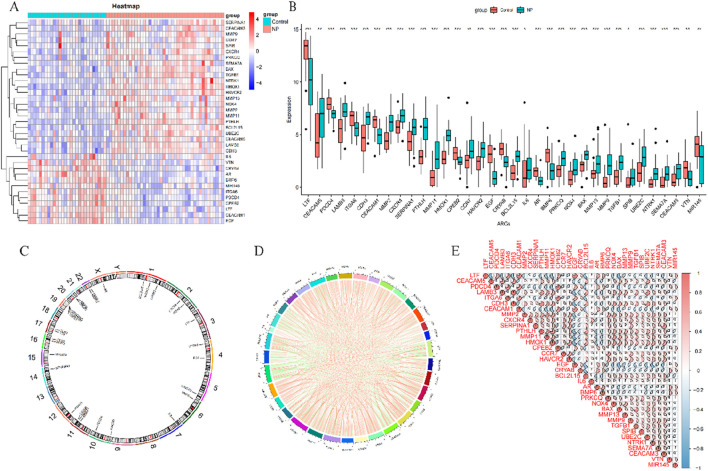
Expression profiles of ARGs in CRSwNP **(A)** Heat map showing the expression of 35 differentially expressed ARGs; **(B)** Box polt showing the exprssion differences of 35 ARGs between CRSwNP and control samples; **(C)** Relative positions of the 35 ARGs on the chromosomes; **(D)** Correlation circle plot showing the correlation of the 35 differentially expressed ARGs; **(E)** Correlation heat map showing that the correlation coefficients of the 35 differentially expressed ARGs. Red and blue represent positive and negative correlations, respectively. Correlation coefficients are shown as the area of the pie chart. *p < 0.05, **p < 0.01, ***p < 0.001.

To explore the role of ARGs in CRSwNP, we conducted a systematic assessment of their expression in CRSwNP patients using the dataset GSE136825. Our analysis identified variations in the expression levels of 35 ARGs. Notably, 23 ARGs (*CEACAM5, LAMB3, CDH3, MMP2, CXCR4, SERPINA1, PTHLH, MMP11, HMOX1, CCR7, HAVCR2, BCL2L15, PRKCQ, NOX4, BAX, MMP13, MMP9, TGFB1, SPIB, UBE2C, NTRK1, SEMA7A, CEACAM3*) were upregulated, while 12 ARGs (*LTF, PDCD4, ITGA6, CEACAM1, CPEB2, EGF, CRYAB, IL6, AR, BMP6, VTN, MIR145*) showed downregulation (Figures 2A,B). Moreover, we plotted the chromosomal locations of these 35 ARGs (Figure 2C) and conducted correlation analysis to explore their interactions, revealing significant associations among specific anoikis regulatory genes (Figures 2D,E).

### 3.2 Immune profile of CRSwNP patients and its association with ARGs

**FIGURE 3:**
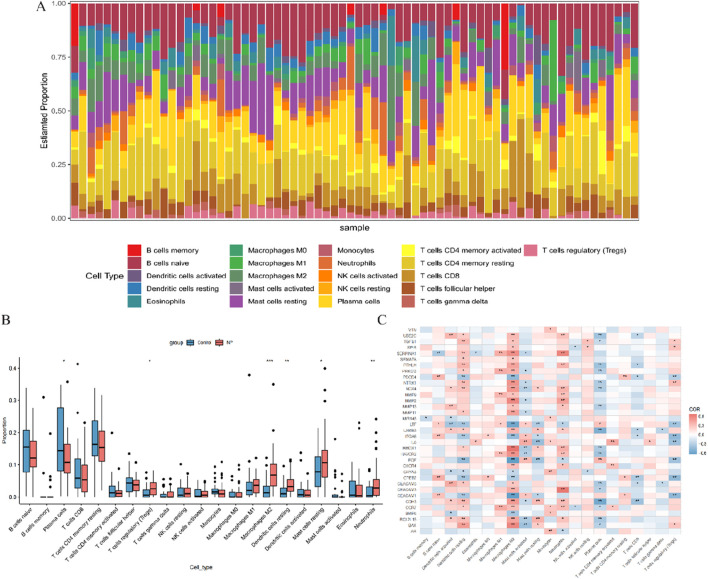
Overview of immune infiltration in CRSwNP **(A)** Relative abundance of 22 infiltrating immune cells between CRSwNP and control samples; **(B)** Box plot showing the difference in immune infiltration between CRSwNP and control samples; **(C)** Correlation analysis of 35 differentially expressed ARGs with infiltrating immune cells. *p < 0.05, **p < 0.01, ***p < 0.001.

The CIBERSORT algorithm, based on gene expression analysis, was employed to evaluate the variation in the proportions of 22 infiltrating immune cell types in each sample. The results indicated a significant upregulation of regulatory T cells (Tregs), M2 macrophages, resting dendritic cells, resting mast cells, activated mast cells, and neutrophils in patients with CRSwNP, while plasma cells showed a downregulation (Figures 3A,B). These findings suggest a substantial impact of CRSwNP on the immune system. Additionally, correlation analysis revealed strong associations among activated dendritic cells, resting dendritic cells, M2 macrophages, activated mast cells, neutrophils, plasma cells, Tregs, and anoikis regulators (Figure 3C), indicating a significant role of ARGs in the immune infiltration alterations observed in CRSwNP patients.

### 3.3 Identification of anoikis-related clusters in CRSwNP

**FIGURE 4:**
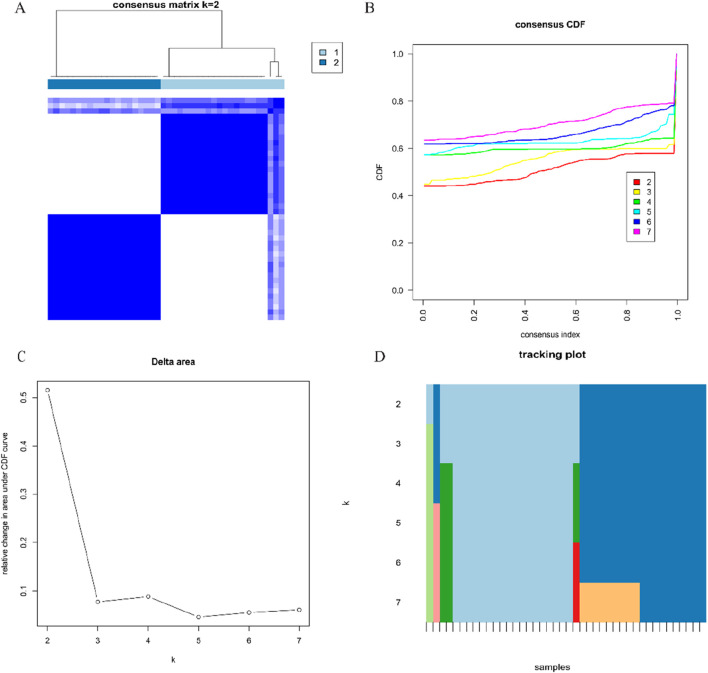
Identification of molecular clusters associated with anoikis in CRSwNP **(A)** Consensus clustering matrix at k = 2; **(B)** Cumulative distribution function (CDF) curves representing k values of 2–7, respectively; **(C)** Representative CDF delta area curves; **(D)** Tracking plot for k values of 2–7, respectively.

To provide a more comprehensive depiction of the expression profile of ARGs in CRSwNP, we applied the consensus clustering algorithm to classify 42 CRSwNP samples based on the expression patterns of 35 ARGs. By varying the value of k from 2 to 7, we found that at k = 2, the consensus index of the cumulative distribution function (CDF) curve displayed minimal fluctuations within a narrow range, accompanied by a notably high consensus score, indicating the optimal value of k to be 2 (Figures 4A–D). Additionally, principal component analysis (PCA) revealed significant distinctions between the two clusters. As a result, we stratified the 42 CRSwNP patients into two distinct clusters.

### 3.4 Identification of immune microenvironment and biological function characteristics in different anoikis clusters

**FIGURE 5:**
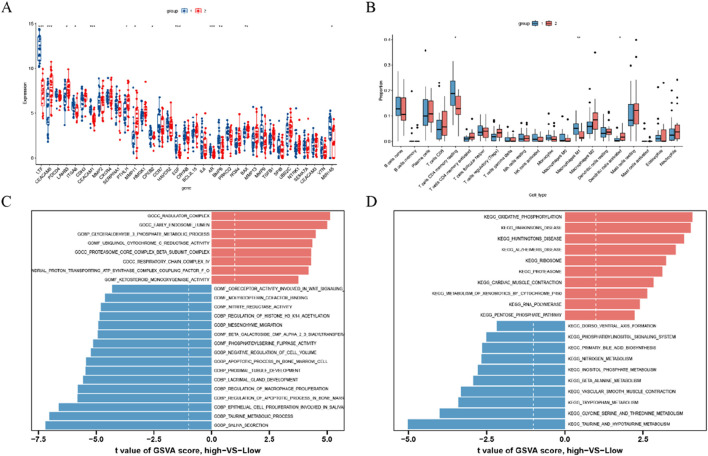
Identification of immune infiltration and biological functional characteristics in different clusters of anoikis **(A)** Boxplot showing the difference in expression of 35 anoikis -associated DEGs between two anoikis clusters; **(B)** Boxplot showing the difference in immune infiltration between two anoikis clusters; **(C)** GSVA results of the GO set between two anoikis clusters were plotted in the bar graph; **(D)** GSVA results of the KEGG gene set between two anoikis clusters are plotted in the bar graph. *p < 0.05, **p < 0.01, ***p < 0.001.

We examined the expression of 35 DEGs between two clusters and identified distinct patterns. Cluster 1 demonstrated high expression levels of *LTF, CEACAM1, CPEB2, EGF, AR, BMP6*, and *MIR145*, whereas cluster 2 exhibited elevated levels of *CEACAM5, LAMB2, PTHLH, MMP11*, and *BAX* (see Figure 5A). Furthermore, to assess variations in immune microenvironment characteristics between the distinct anoikis-associated clusters, we analyzed differences in infiltrating immune cells and their functions. Our findings indicate that cluster 1 was enriched with resting memory CD4^+^ T cells and M1-polarized macrophages, while cluster 2 showed a higher proportion of activated dendritic cells (Figure 5B), suggesting a unique immune profile between these clusters.

Subsequently, we conducted GSVA based on GO and KEGG gene sets. The GO analysis revealed significant upregulation of pathways including the ragulator complex, early endosome lumen, glyceraldehyde-3-phosphate metabolic process, ubiquinol cytochrome C reductase activity, proteasome core complex beta subunit complex, respiratory chain complex IV, mitochondrial proton-transporting ATP synthase complex coupling factor F O, and ketosteroid monooxygenase activity in cluster 2. Conversely, pathways related to the regulation of macrophage proliferation, mesenchyme migration, saliva secretion, taurine metabolic process, epithelial cell proliferation involved in salivary, and regulation of apoptotic process in bone marrow showed downregulation in cluster 2 (Figure 5C). The KEGG analysis revealed that pathways such as oxidative phosphorylation, Parkinson’s disease, Huntington’s disease, Alzheimer’s disease, ribosome, proteasome, cardiac muscle contraction, metabolism of xenobiotics by cytochrome P450, RNA polymerase, and pentose phosphate pathway were upregulated in cluster 2. On the other hand, pathways including taurine and hypotaurine metabolism, glycine, serine and threonine metabolism, tryptophan metabolism, vascular smooth muscle contraction, beta-alanine metabolism, inositol phosphate metabolism, glycerophospholipid metabolism, homologous recombination, systemic lupus erythematosus, metabolism of xenobiotics by cytochrome P450, and drug metabolism cytochrome P450 were downregulated in cluster 2 (Figure 5D).

### 3.5 Construction and validation of the lasso model, SVM model, and RF model

**FIGURE 6:**
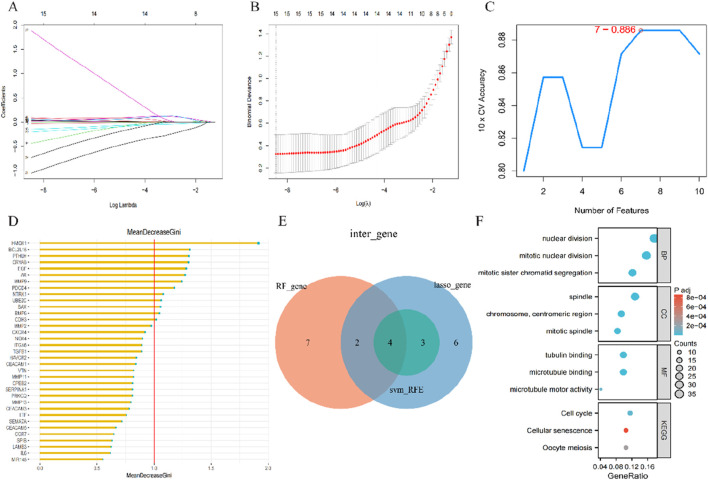
Construction and validation of the Lasso model, SVM model and RF model **(A)** Fourteen cross-validations of adjusted parameter selection in the LASSO model. Each curve corresponds to one gene; **(B)** LASSO coefficient analysis. Vertical dashed lines are plotted at the best lambda; **(C)** SVM-RFE algorithm for feature gene selection; **(D)** RF algorithm for feature gene selection; **(E)** Venn diagram showing the feature genes shared by LASSO, SVM-RFE algorithms, and random forest **(F)** Bubble plot of GO and KEGG analysis results based on the 4 feature genes.

We utilized three algorithms to identify candidate genes associated with anoikis from a pool of 35 DEGs related to anoikis for predicting the occurrence of CRSwNP. The lasso model results indicated that 14 genes were linked to the occurrence of CRSwNP, including *LTF, PDCD4, CDH3, CXCR4, SERPINA1, PTHLH, CPEB2, CRYAB, BCL2L15, AR, PRKCQ, TGFB1, SPIB, SEMA7A*, and *VTN* (Figures 6A,B). In parallel, support vector machine (SVM) feature vectors were generated, identifying 7 genes as anoikis variables: *PTHLH, PDCD4, SEMA7A, CDH3, SERPINA1, AR*, and *VTN* (Figure 6C). Using the random forest algorithm, we identified 13 signature genes with importance scores exceeding two, including H*MOX1, BCL2L15, PTHLH, CRYAB, EGF, AR, MMP9, PDCD4, NTRK1, UBE2C, BAX, BMP6*, and *CDH3* (Figure 6D). Finally, by intersecting the genes obtained from the three machine learning models, we identified 4 hub genes (*PDCD4, CDH3, PTHLH*, and *AR*) for further analysis (Figure 6E). The Gene Ontology (GO) and Kyoto Encyclopedia of Genes and Genomes (KEGG) results indicated that these genes were predominantly involved in nuclear division, spindle organization, tubulin binding, and cell cycle regulation (Figure 6F).

**FIGURE 7:**
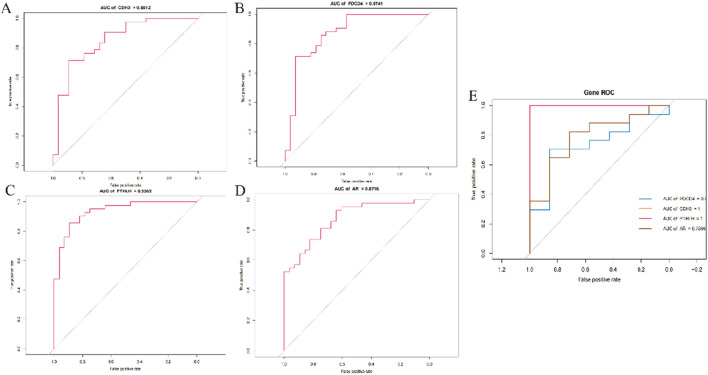
Exploration of the diagnostic value of 4 signature genes **(A)** ROC curves showing the diagnostic value of the CDH3 gene in the GSE136825 dataset; **(B)** ROC curves showing the diagnostic value of the PDCD4 gene in the GSE136825 dataset; **(C)** ROC curves showing the diagnostic value of the PTHLH gene in the GSE136825 dataset; **(D)** ROC curves showing the diagnostic value of the AR gene in the GSE136825 dataset; **(E)** ROC curves showing the diagnostic value of the 4 signature genes in the GSE179265 dataset.

Following this, we conducted receiver operating characteristic (ROC) analysis and calculated the area under the curve (AUC) values to assess the accuracy of each diagnostic gene. Our results showed that all 4 key genes demonstrated relatively high predictive values in the training set (GSE136825; Figures 7A–D). Additionally, we validated these findings in another dataset (GSE179625; Figure 7E).

### 3.6 Establishment of nomogram for predicting CRSwNP

**FIGURE 8:**
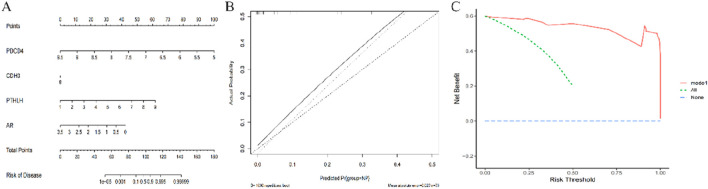
Construction of the nomogram model based on Characteristic ARGs **(A)** Construction of nomogram integrating Characteristic ARGs for CRSwNP. in the nomogram, each variable corresponds to a score, and the total score can be calculated by summing the scores of all variables; **(B)** Calibration curves to estimate the prediction accuracy of the nomogram; **(C)** Decision curve analysis showing the clinical benefit of nomogram.

A nomogram was developed for CRSwNP based on four key genes (Figure 8A). Each key gene in the nomogram is assigned a score, and the total score is calculated by summing the scores of all the characteristic genes. This total score is indicative of varying risks associated with CRSwNP. Calibration curves confirmed the accuracy of the nomogram in predicting the onset of CRSwNP (Figure 8B). Decision curve analysis illustrated the potential benefits of using the nomogram for patients with CRSwNP (Figure 8C).

### 3.7 Immune infiltration correlation analysis of 4 key genes

**FIGURE 9:**
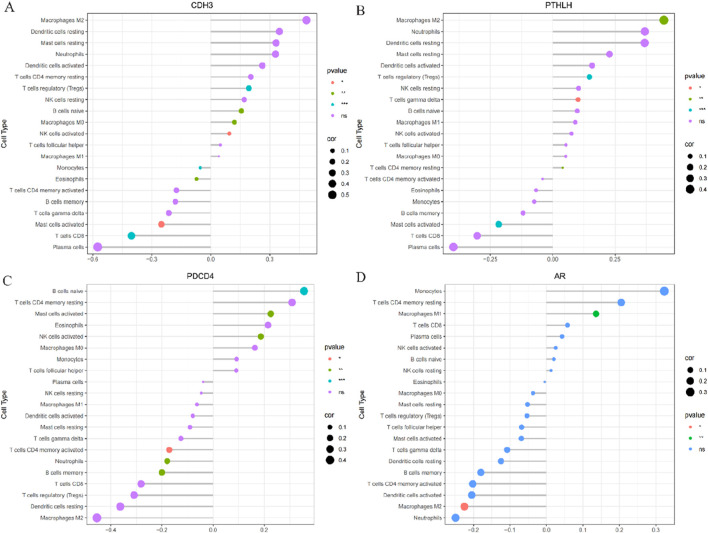
Correlation analysis of immune infiltration with signature gene expression in the combined database of GSE136825 database and GSE1719265 database **(A)** The correlation of CDH3 gene expressions with immune infiltration cell; **(B)** The correlation of PTHLH gene expressions with immune infiltration cell; **(C)** The correlation of PDCD4 gene expressions with immune infiltration cell; **(D)** The correlation of AR gene expressions with immune infiltration cell. The size of the dots represents the strength of gene correlation with immune cells; the larger the dot, the stronger the correlation. The color of the dots represents the p-value.

The correlation between gene expression and immune cell infiltration of the four hub genes was analyzed in the combined database of GSE136825 and GSE179625. The results indicated a positive correlation of the AR gene with Macrophages M1 and a negative correlation with Macrophages M2 (Figure 9A). The CDH3 gene exhibited positive correlations with T cells regulatory (Tregs), B cells naive, Macrophages M0, and NK cells activated, and negative correlations with T cells CD8^+^, Mast cells activated, Eosinophils, and Monocytes (Figure 9B). PDCD4 gene showed a positive correlation with B cells naive, Mast cells activated, and NK cells activated, while PDCD4 gene was negatively correlated with T cells CD4^+^ memory activated, Neutrophils, and B cells memory (Figure 9C). The PTHLH gene displayed positive correlations with Macrophages M2, T cells regulatory (Tregs), T cells gamma delta, and T cells CD4^+^ memory resting, whereas the PTHLH gene showed a negative correlation with Mast cells activated (Figure 9D). These findings suggest that the expression of these genes may influence the levels of immune cell infiltration, indicating their potential involvement in immune regulation in the pathogenesis of CRSwNP.

### 3.8 Construction of regulatory networks

**FIGURE 10:**
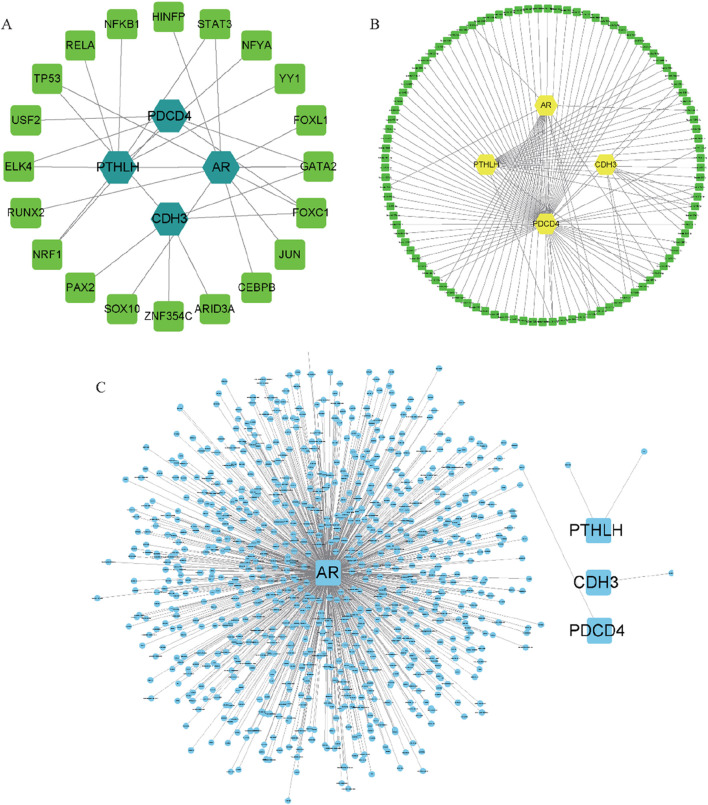
Construction of regulatory network and Drug-gene interaction network **(A)** 4 target gene-miRNA regulatory network. Green-blue hexagon nodes represent hub genes, green squares represent miRNAs; **(B)** 4 target gene-TF regulatory network. Yellow hexagon nodes represent hub genes, green squares represent TFs; **(C)** Drug-gene interaction network. Blue squares nodes are pivotal genes and blue circle nodes are predicted drugs or molecular compounds.

Subsequently, we constructed the gene-miRNA regulatory network (Figure 10A) and gene-transcription factor (TF) regulatory network (Figure 10B) for the four key genes, respectively. The results revealed the intricate involvement of multiple miRNAs and TFs in regulating these pivotal genes. Furthermore, a drug interaction regulatory network for AR was developed (Figure 10C). Collectively, these findings provide valuable insights into the prospective utilization of these genes in disease diagnosis, subtype classification, survival prognosis, drug responsiveness assessment, and other pertinent domains.

### 3.9 Experimental verification

**FIGURE 11:**
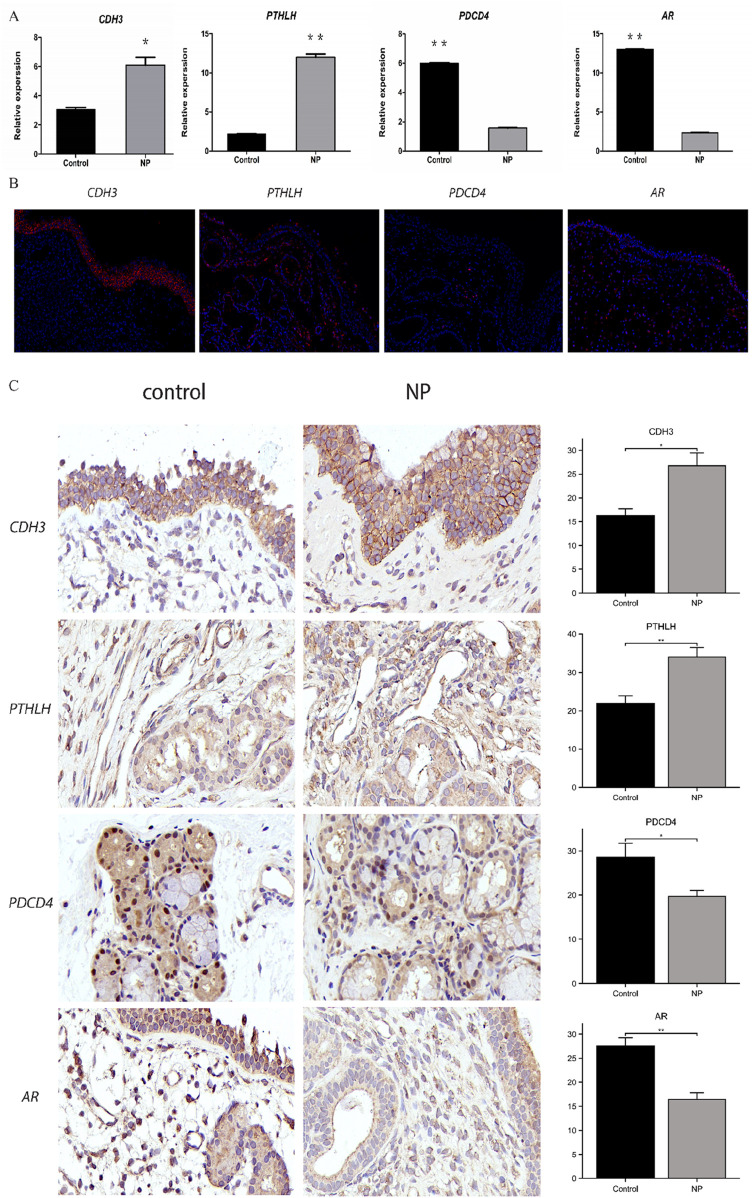
The expression of the identified 4 hub genes (CDH3, PTHLH, PDCD4 and AR) (N = 3) **(A)** mRNA expression of hub genes from human’s nasal polyps samples by qRT-PCR verification. **(B)** Immunofluorescence staining of consecutive serial sections of hub genes from human’s nasal polyps samples.Original magnification ×40. **(C)** Immunohistochemical staining of consecutive serial sections and H-Score of hub genes from human’s nasal polyps samples and control samples. Original magnification ×100. *P < 0.05; **P < 0.01; ***P < 0.001. Image magnification is 100×.

Finally, the potential targets mentioned previously were validated using qRT-PCR (Figure 11A), IF (Figure 11B) and IHC (Figure 11C). As depicted in the figures, mRNA and protein levels of *PTHLH* and *CDH3* were notably elevated in nasal polyp tissue compared to the control group, whereas *PDCD4* and *AR* were downregulated (*P* < 0.05). These experimental findings align with our collected data.

## 4 Discussion

CRSwNP presents a significant clinical challenge in nasal surgery due to its high recurrence rate, which adversely affects patients’ quality of life and places a substantial burden on society. Researchers have long been committed to exploring innovative diagnostic methods and therapeutic strategies to improve the diagnosis and treatment of CRSwNP. Apoptosis, the most common form of programmed cell death in multicellular organisms, can be triggered by either intrinsic or extrinsic pathways (Christgen et al., 2022). Anoikis, essentially an apoptotic process, can result from an intrinsic pathway mediated by mitochondria or an extrinsic pathway activated by cell surface death receptors (Taddei et al., 2012). However, further exploration is needed to understand the specific mechanisms through which anoikis regulates diseases. In this study, we employed three machine learning algorithms to examine the role of ARGs in CRSwNP. For the first time, we conducted a comprehensive analysis of the differential expression profile of ARGs between normal nasal samples and nasal polyp samples. Our findings revealed 35 dysregulated ARGs in CRSwNP patients, indicating the potential involvement of anoikis in the development of CRSwNP.

Considerable research has shown that CRSwNP is characterized by the upregulation of various immune cells such as monocytes, macrophages, dendritic cells, neutrophils, mast cells, eosinophils, basophils, and ILC2s, accompanied by the downregulation of Treg cells (Lei et al., 2022; Laidlaw and Buchheit, 2020). Different immune cell types play crucial roles in the pathogenesis of CRSwNP. Mast cells, for example, secrete a plethora of cytokines like IL-5, Granulocyte Macrophage Colony-Stimulating Factor, eotaxin, and RANTES, which activate eosinophils. This activation leads to tissue remodeling, chymase expression inducing mucus secretion, and the release of mediators causing tissue edema. Furthermore, mast cells release preformed mediators and newly synthesized proinflammatory molecules, potentially contributing to nasal polyp development (Takabayashi et al., 2012; Pawankar, 2005). Eosinophils worsen nasal polyp severity by producing toxic cationic proteins damaging epithelial cells and releasing proinflammatory molecules promoting type 2 inflammation (Martin et al., 1996; Seiberling et al., 2012). They also sustain plasma cells and antibody production in the bone marrow long-term and activate T cells during inflammation (Chu et al., 2011; Jacobsen et al., 2012). Disease-specific dendritic cell specialization may influence and perpetuate distinct polarized T cell responses in eosinophilic and non-eosinophilic CRSwNP. Increased expression of Thymic Stromal Lymphopoietin (TSLP) and osteopontin in CRSwNP correlates with dendritic cells’ ability to induce Th2 and Th1/Th17 responses, respectively (Cao et al., 2016; Shi et al., 2014). TSLP-stimulated dendritic cells drive naïve CD4^+^ T cells to differentiate into Th2 cells in an OX40-dependent way (Ito et al., 2005) and secrete high levels of CCL17 and CCL22, recruiting Th2 cells (Liao et al., 2015). Osteopontin has been shown to promote Th1 and Th17 cell differentiation by inducing IL-12, IL-6, and IL-23 production from dendritic cells (Kourepini et al., 2014). M1 macrophages produce pro-inflammatory cytokines leading to tissue damage but aiding immune clearance, while M2 macrophages mainly secrete anti-inflammatory cytokines, supporting wound healing and tissue repair (Sica and Mantovani, 2012; Murray et al., 2014; Martinez and Gordon, 2014). Imbalance in M1/M2 macrophage homeostasis may result in impaired phagocytic function in type 2 inflammation seen in CRSwNP (Lan et al., 2019). Increased CCL25 expression by M1 macrophages in CRSwNP might contribute to nasal polyp formation. The positive association of CD4^+^ T cells in CRS may explain the severity of inflammation (Seif et al., 2018).Clustering analysis revealed that CRSwNP patients could be categorized into two clusters, with cluster 1 showing higher levels of CD4^+^ memory resting T cells and M1 macrophages. Therefore, patients in cluster 1 may have a poorer prognosis, and targeting different immune cells in CRSwNP treatment strategies may be crucial for future research.

In recent years, machine learning has become increasingly prominent in the diagnosis of Chronic Rhinosinusitis with Nasal Polyps (CRSwNP), screening of key genes, and assessment of immune cells, attributed to its superior predictive performance, lower error rates, and higher reliability (Dunn and Rothenberg, 2022; Ramakrishnan et al., 2021). The study utilized LASSO, SVM-RFE, and RF algorithms to identify four signature genes: PDCD4, CDH3, PTHLH, and AR. These four genes exhibited strong diagnostic value with all Area Under the Curve (AUC) values exceeding 0.85 in the training set (n = 42). Their diagnostic performance in the validation set was also promising, with all AUC values surpassing 0.739. Notably, CDH3 and PTHLH attained AUC values of 1, potentially influenced by the limited sample size of nasal polyps (NP) in the validation group (n = 27). Additionally, a nomogram incorporating these four genes was developed to enhance their diagnostic potential for improved identification of CRSwNP onset.

Simultaneously, several studies have revealed the involvement of key diagnostic genes in the pathogenesis of CRSwNP. PDCD4, a target of miR-21, functions by inhibiting cytokine expression through the miR-21/PDCD4/NF-κB pathway, which ultimately suppresses inflammation in CRSwNP (Liu et al., 2021).

AR, which is located on the X sex chromosome, encodes the androgen receptor, which is a nuclear transcription factor.In myocarditis, it was found that inhibition of androgen receptor expression reduced the production of cytokine signal inhibitors (SOCS3, SOCS1) and enhanced the activation of signal transduction and transcriptional activators (STAT3, STAT5), resulting in M2-type polarization of macrophages (Brescia et al., 2022; Ramos et al., 2022). Moreover, the AR, acting as a nuclear transcription factor receptor, exhibits distribution throughout nasal polyps. Another study revealed that suppression of androgen receptor (AR) attenuates sepsis-induced acute lung injury (ALI) by inhibiting M1 macrophage polarization and pro-inflammatory cytokine secretion, further validating the regulatory relationship between AR and M1 macrophages (Wang et al., 2025). The study revealed that the stemness maintenance capacity of tumor-infiltrating CD8^+^ T cells governs sexual dimorphism in tumor immunity, while intrinsic androgen receptor (AR) signaling significantly suppresses the properties of stem-like CD8^+^ T cell subsets (Yang et al., 2022). Additionally, CDH3 plays a critical role in cell differentiation, growth, and migration (Gloushankova et al., 2017; Kourtidis et al., 2017), while PTHLH is involved in glandular formation in epithelial systems and serves a role in regulating epithelial–mesenchymal interactions during mammary development and ductal morphogenesis in adolescence (Wu et al., 2011). PTHLH-mediated M2 macrophage enrichment was identified in bladder cancer. Our single-cell analysis further revealed a non-significant but consistent increase of M2 macrophages in Cluster 2, implying PTHLH may contribute to their induction—a hypothesis requiring mechanistic validation (Wang et al., 2022). Parallel studies in renal carcinoma established that the SLC17A9-PTHLH-EMT signaling axis critically drives oncogenic proliferation and invasion (Li et al., 2022).

Our preliminary experimental validation indicated differential mRNA and protein expression of these key genes. According to the protein localization results, CDH3, functioning as a cadherin, is predominantly expressed at the junctions of nasal mucosal epithelial cells. PTHLH, a secreted protein, is distributed across all layers of nasal polyps, and PDCD4 is primarily localized in the cytoplasm and nucleus of glandular epithelium within nasal polyps. In conclusion, the investigation of these characteristic genes provides some degree of support for the credibility of our screening outcomes.

Gene enrichment analysis indicated that the key genes identified were predominantly linked to IGF1 receptor signaling pathways, regulation of epithelial cell proliferation, regulation of transforming growth factor β2 production, regulation of integrin biosynthetic processes, and epithelial-to-mesenchymal transition involved in cardiac fibroblast development, among other pathways. Nevertheless, further experimental validation is needed to elucidate the regulatory connections between these key genes and the underlying mechanisms of various signaling pathways in patients with CRSwNP.

Our study entailed a comprehensive examination of these central genes, focusing on analyzing their association with immune infiltration and investigating their interaction networks involving miRNAs, transcription factors (TFs), and drug regulations. Through this analysis, we aim to inform our future approaches in targeting and immunotherapy for CRSwNP. Our next step involves further investigating the potential molecular mechanisms of these genes in CRSwNP through molecular biology experiments.

This study has several limitations. First, the patient population and selection criteria in the public database datasets present certain constraints. To mitigate potential biases from dataset selection, we utilized the dataset with the most comprehensive patient information. Second, the impact of geographical or ethnic variations on gene expression profiles remains unclear. Additionally, relevant clinical data—such as age, gender, and disease duration—which may influence gene expression, were not incorporated. Finally, the specific mechanisms through which *PDCD4*, *AR*, *CDH3*, *and PTHLH* regulate immune cell infiltration and affect disease progression require further in-depth investigation.

## 5 Conclusion

We identified two anoikis-related clusters in CRSwNP and discovered four key genes associated with CRSwNP, in which AR was highly expressed in cluster 1 and PTHLH was highly expressed in cluster 2. These findings may provide new insights for drug screening, personalized therapy, and immunotherapy strategies for CRSwNP.

## Data Availability

The datasets presented in this study can be found in online repositories. The names of the repository/repositories and accession number(s) can be found in the article/supplementary material. Sequence data that support the findings of this study have been deposited in the European Nucleotide Archive with the primary accession code PRJNA563822 and PRJNA744406.

## Abbreviations

CRSwNP, Chronic rhinosinusitis with nasal polyps; ARGs, anoikis-related genes; LASSO, least absolute shrinkage and selection operator; SVM, support vector machine-recursive feature elimination; RF, random forest; ROC, receiver operating characteristics; GO, gene ontology; KEGG, Kyoto Encyclopedia of Genes and Genomes; TF, transcription factor; ECM, extracellular matrix; EGFR, epidermal growth factor receptor; ROS, reactive oxygen species; MMPs, matrix metalloproteinases; TIMPs, tissue inhibitors of metalloproteinases; DEGs, differentially expressed genes; ML, machine learning; AUC, Area under the ROC curve; CIBERSORT, Estimating Relative Subsets Of RNA Transcripts; GSVA, Gene Set Variation Analysis; DC, dendritic cell; NPs, nasal polyps; Tregs, T cells regulatory.
